# Describing digital nursing work in a remote patient monitoring application: Novel convergent mixed methods secondary analysis of feasibility trial data

**DOI:** 10.1177/20552076261462734

**Published:** 2026-06-18

**Authors:** Rosalynn C. Austin, Bjørg Karlsen, Ingvild Morken, Sara Sudqvist, Aurora Selvik, Anne Marie Lunde Husebø, Hege B. Wathne

**Affiliations:** 1 Department of Public Health, Faculty of Health Sciences, 56627 University of Stavanger, Stavanger, Norway; 2 Department of Cardiology, Portsmouth Hospitals, University NHS Trust, Portsmouth, UK; 3 Long Term Conditions Theme, National Institute of Health and Care Research (NIHR) Applied Research Collaborative (ARC) Wessex, Southampton, UK; 4 Department of Quality and Health Technologies, Faculty of Health Sciences, 56627 University of Stavanger, Stavanger, Norway; 5 Research Group of Nursing and Health Sciences, Research Department, Stavanger University Hospital, Stavanger, Norway; 6 Dignio AS, Stenersgata, Oslo, Norway; 7 Department of Caring and Ethics, Faculty of Health Sciences, 56627 University of Stavanger, Stavanger, Norway

**Keywords:** nursing care, digital health, remote patient monitoring, chronic illness, convergent mixed methods

## Abstract

**Objectives:**

Despite the prevalence of applications which offer remote patient monitoring (RPM) in chronic illness management, the use of RPM in healthcare systems remains low. There is limited use of data generated in an RPM, to describe the work of clinicians. The study’s aim was the use a novel evaluation method to characterise the nurse work in an RPM intervention.

**Methods:**

A convergent mixed methods design was used to perform secondary analysis on feasibility trial data. Data sets: (1) RPM data set: data generated by usage, and (2) Interview data set: nurse user interviews. In the RPM data set, patient entered data, software data labelling, and nurse responses were defined as data strings. Quantitative variables were summarized using descriptive statistics. Both data sets were analysed using systematic text condensation. In the RPM data set themes were translated into quantitative data enabling reportable links to RPM functionality. Themes converged and narrative integration triangulated the data.

**Results:**

Seven patients (heart failure (n=4); colorectal cancer (n=3)) and eight nurses were selected. Their RPM intervention engagement generated a mean of 97 data strings. Converging themes included: (1) intervention technical and operational work, (2) digitally enabled care management and coordination, and (3) educational and relational work.

**Conclusions:**

The evaluation method provided a detailed characterization of the digital nurse work performed in the RPM intervention. While data presented is foundational, it highlights the potential of how evaluation of nurse engagement with RPM application may provide essential feedback to refine digital interventions to ensure efficient integration in healthcare systems.

## Plain Language Summary

**Aims of purpose of the research:**

Our study looked at how nurses use remote patient monitoring (RPM) app to support people with long-term health conditions. Our goal was to better understand the types of work nurses do in digital health interventions to improve the design of future RPM systems.

**Background of the research:**

There are lots of new RPM apps being created, but the use of them in healthcare systems outside of research studies is limited. Rarely is the data generated using apps used to describe the work of nurses around such digital health tools.

**Methods and research design:**

We re-analysed data from a feasibility study of an RPM intervention. We combined two types of information: data automatically recorded by the RPM app when patients and nurses used it and interviews from the nurses. We called each occurrence of patient-entered information, app labels and nurse responses as a “data string”. We used both simple counts and detailed text analysis to describe the work nurses did around the app.

**Results and importance:**

Seven patients and eight nurses took part and on average generated 97 data strings per patient. By combing the data, we observe three types of digital nursing work: technical and operational tasks to keep the intervention running; digitally enabled care management and coordination; and educational and relational work to support patients. Our new way of evaluating digital health applications provided clearer pictures of the work nurses do around an RPM app. These findings are early stage, but they show that analysing detailed usage data together with nurse interviews can give important feedback which will improve digital health tools. These improvements will better support nurses and increase adoption in healthcare services.

## Background

The use of digital health technologies integrated into patient pathways is on the rise. One of the promising uses is that of remote patient monitoring (RPM). RPM includes a wide variety of non-invasive and invasive tools to monitor people with chronic illnesses.^1^ RPM applications have two users; healthcare professionals (frequently nurses) and patients.^2,3^ These applications include tracking clinical measurements (e.g., heart rate, blood pressure, weight, etc.), patient reported subjective symptoms and quality of life levels, health/illness education modules, communication portals, and application notifications.^2–5^ RPM applications are being targeted as key tools for the management of people with chronic illnesses following a hospitalization or as prevention to a hospital admission.^4–7^ Chronic illness is any non-communicable illness where long-term illness management is the goal.^8^ Illness management for patients requires they assume the responsibility for complex self-management regimens at home.^9^ While eHealth solutions are viewed as promising solutions to overwhelmed healthcare systems,^3,10^ the evidence supporting their effect on mortality and readmissions is varied.^11^ RPM research has the potential to reduce hospitalizations and strain on the healthcare system,^2,3,12^ however the impact RPM applications have on clinical workload is not well understood.^13^

RPM applications are dependent on meaningful user engagement to ensure effective communication between patients and nurses.^14^ Engagement with RPM means additional work needs to be completed by users (patients and nurses). Typical definitions of RPM user engagement are patient focused and include: (1) application usage patterns,^15^ (2) describing the usability, accessibility, and application features,^16^ and (3) alterations of patient health behaviours.^17^ The rise of nurse assisted RPM applications may have the ability to provide supportive care for those with chronic illnesses.^18^ For nurses, engagement with RPM platforms means a new type of work, that of digital remote assessment.^14^ Description of this work has focused on the time spent and actions completed on the RPM.^15–17^ Evidence Based Health Informatics, promoted by the International Medical Informatics Association, has called for robust evaluations of eHealth solutions including describing user engagement.^3,10^ The evaluation of the clinical users of RPM applications such as nurses has not been frequently reported, and much is unknown about the best way to perform this evaluation.^3,10^

Despite the promise of the use of RPM in chronic illness management the implementation and integration into healthcare services is slow.^19^ The Norwegian Helse Vest digital strategy acknowledges the need for research which targets the integration of digital solutions in existing healthcare pathways.^20^ Most existing research on the impact of RPM applications on healthcare professionals’ workloads identified barriers to eHealth uptake in healthcare services^2^ and explored health economic evaluations.^11,21^ A qualitative literature review reports on the alterations to clinical workload and decision making relating to RPM clinical work^19^ and a recent cross-sectional study of nurses in the Netherlands outlined digital nursing work and its intensity in virtual care centres.^22^ There is scant evaluation of healthcare professionals’ (including nurses) engagement with RPM applications.^3,10,11^ To enable successful implementation of RPM in healthcare services there is a need for more evaluation of the digital work of healthcare professionals (typically nurses) involved in RPM applications, including the impact on clinical workload, additional skills, and decision-making.^3,10,19,23,24^ This paper will address a critical gap in evaluation of digital health solutions^25^ and may provide insight into ways to address the increased nurse workload that is a barrier to digital health adoption.^24^

### Prior work

The eHealth@Hospital-2-Home (eHealth@H2H) research project, funded by the Norwegian Research Council (NRC ID 301472) was designed to develop and test an RPM application tailored to patients with specific chronic illnesses: heart failure and colorectal cancer. The RPM was used in two different Norwegian hospitals.^7^ The RPM platform (Dignio) had two interfaces, one for patients (My Dignio) and one for nurses (Dignio Prevent). RPM patients were “prescribed a dose” of engagement tasks (input daily clinical and symptom measurements, weekly quality of life measurements). Other features (illness specific educational lessons, text messaging, or video conferencing with nurses) were designed as optional patient discretion components. The nurses (nurse navigators) were instructed to monitor data imputed and respond to application notifications (technical and clinical).^7^ The data imputed by patients created notifications flags for the nurses to manage and respond to. The notifications had different purposes and colours: Black – personalized patient safety parameters not set by clinical user, Blue – new message from patient, Green – data point missing, Yellow – moderate safety limit warning, Red – severe safety limit warning. Some flags (safety – yellow and red) had the ability for clinical users (nurses or supporting doctors) to personalize to the patients. Nurses were asked to respond to safety flags (once seen) within a week (yellow) or 24-48 hours (red). Patients were instructed that the system was not under 24-hour surveillance and that they should seek care through standard pathways if they felt unwell. The feasibility study evaluated the user experience and acceptability of the RPM intervention for the patients and nurse navigators.^6^ The RPM application was reported to improve communication between nurses and patients and nurses reported observed improvements in patients’ chronic illness management.^14^

### Objectives

**Aim:** The aim of this study was to use novel analysis method^26^ and report on the findings of this method using data from a feasibility study that used the Dignio RPM platform. The overarching **research question** was asked: Can the use of automatically generated data from user engagement (i.e., patient and nurse engagement) with an RPM intervention describe the work nurses performed during the nurse-assisted RPM intervention conducted in the eHealth@H2H feasibility study?

## Methods

### Design

A convergent mixed methods design (QUAN + QUAL) was used. In this approach, quantitative and qualitative data were collected and analysed at the same time to determine whether the findings from each data set converge with or diverge from the other.^27^ The quantitative and qualitative analysis received equal weight and enabled the research team to supplement the weaknesses of one method with the strengths of the other. The datasets included quantitative data generated from the routine use of the eHealth@H2H intervention by both patients and nurses in the feasibility study (RPM dataset) and interview data from the nurses who provided the clinical monitoring in the feasibility study (interview dataset). The datasets were integrated to provide in-depth data around the nurse experience in RPM.

### Data sampling and analysis

**Feasibility study sampling:** Eligibility and recruiting sites were previously reported.^28^ Participants from that study were purposively sample to ensure representation across both recruiting sites in Norway, both chronic illness populations, genders and ages. This also ensured good representation of nurse users to generate the RPM dataset.

**RPM dataset sampling and analysis:** A purposive sample of patient participants (n =7), with associated nurses (n=8) from the feasibility study were selected to ensure representation from both recruiting sites, illness pathologies (heart failure and surgical colorectal cancer patients), genders, throughout the duration of the feasibility study. The maximum variability in the sample was chosen to enable different types of engagement with the platform which would enable an early assessment of this analysis method to provide insight into user engagement.

**Figure 1.**
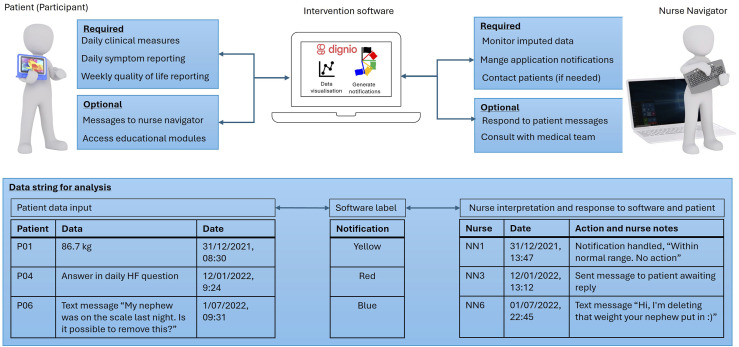
Visiualisation of data string(s) extracted for analysis, with exemplar data strings. Data strings were created by user (patient and nurse) engagement with remote patient monitoring intervention software (My Dignio and Dignio Prevent). 3D human model images from PixaBay^29^ by artist “peggy_marco-1553824”.

The RPM dataset was a mixture of quantitative (e.g., clinical measurements, times of data entry, etc.) and qualitative data (e.g. text messaging between patients and nurses, nursing notes, notifications, etc.) that was qualitatively coded before being translated back into quantitative data to facilitate description of work performed. Through user engagement, the software program created application notifications to alert the nurses of events required their attention had occurred. The software program also provided the nurses with the opportunity to add notes to those notifications or create independent nursing notes. User engagement with the app created multiple data strings per data input which was extracted for analysis (Figure 1). My Dignio creates a log of those data strings enabling the research team to extract that data for analysis. Additional material 1 (file name: SupplementalMaterial1_RPM_Dataset_Codebook) has exemplar data strings together with coding and exemplar quotes.

**Quantitative analysis:** The number and type of data strings and notifications were summarized using descriptive statistics. Nurse response time to notifications was calculated and summarized using descriptive statistics. **Qualitative analysis:** Each data string was individually analysed using Malterud^30^ systematic text condensation method to qualitatively describe the nurse work categories and resultant nurse actions. Preliminary themes were labelled as nurse work categories. Sub-themes were labelled as nurse actions and outcomes based on researcher interpretation of the work performed by the nurses based on the available RPM data (additional material 1). Hege B. Wathne (HBW) independently examined the coding and agreed with Rosalynn C. Austin (RCA) coding. **Mixed Method analysis:** These codes were organized into final themes before being translated into quantitative data (counting of the number of data strings for a given theme or sub theme) to describe the proportion of work observed in each theme and by application notifications.^27^ Finally cross tabulations between coding and application notifications were performed in SPSS which characterized of nursing work and actions performed by calculating a percentage of work completed aligned with RPM notification type.

**Interview dataset sampling and analysis:** Secondary analysis,^31^ was performed on interviews conducted with the nurses (n = 8) from the feasibility project. Originally these interviews were a part of a PhD project that explored the acceptability of the Dignio Prevent as experienced by the nurses in the intervention.^14,28^ In the current study, interview data was re-explored in relation to characterizing any work reported around RPM intervention asking the data set three new research questions. Additionally, any work reported by the nurses around patient data on the platform or any work performed that was not related to nursing (e.g., low battery alerts, equipment malfunctions, etc.) was also coded. Nurse participants were not re-interviewed.

Existing interview transcripts were analysed using a thematic analysis approach in a stepwise process, following Malterud’s systematic text condensation.^30^ The nurse interview transcripts were explored around three main research questions: 1) how nurses worked with the data imputed by patients in the digital platform, 2) what was the nurse work around RPM patient consultations, and 3) what RPM application work performed by the nurses was not related to nursing? In the first step, transcripts were reviewed and reread to identify preliminary themes. The second step involved organizing and coding chunks of text that elucidated the research questions. In the third step, the codes were more methodically divided into categories. The fourth and last step, involved summarizing and presenting the findings as themes. Additional material 2 (file name: SupplementalMaterial2_NurseInterview_CodingExamplars) has exemplar data strings together with coding and exemplar quotes.

### Dataset convergence

Following the analysis, RCA and HBW independently examined each other’s coding and analysis. Together they refined and integrated their coding, altering the grouping of sub-themes and meaning units. Through iterative discussion, final themes were agreed on and narrative triangulation of the data occurred to highlight how the quantified observations in the RPM dataset were present in the findings in the interview dataset. This process continued throughout the writing of the results section until both researchers agreed on the presentation of the data and findings presented. A third author (BK) performed an in-depth evaluation of the analysis to confirm and refine the integration of the two datasets. Additional material 3 (file name: SupplementalMaterial3_FinalCodingMap_ThemeCategoryIntegration) provides a final theme and category map with how themes and categories were integrated. Themes will be discussed using an integration of the translated coded data string analysis (RPM data set) and secondary analysis of nurse interviews (interview data set) on their experiences of their work in the RPM intervention. These discussions are for illustrative purposes to highlight the potential ability of this method to prove detailed data-based descriptions of nurse work associated with RPM interventions.

### Ethical approval

The feasibility study was approved by the Norwegian Centre for Research Data (ID.NO: 523386) and the participating hospitals’ Privacy Appeals Board which evaluated privacy measures and compliance with relevant regulations. The Regional Ethics Committee deemed the current feasibility study exempt (ID.NO: 242405). Informed consent and recruitment to the feasibility study (nurses and patients) was previously reported.^14,28^ All participants gave written informed consent, and all findings have been anonymised. The analysis of data presented in this paper, is within feasibility studies approvals and adhered to the Declaration of Helsinki.^32^

## Results

### Participants

In the RPM dataset, seven patients were included (HF; n=4 and CRC; n=3), two were female, and age ranged from 55-85 (Table 1 for details). By selecting these patients, the engagement of seven nurses (nurse navigators; n=6 and a nurse PhD candidate) was captured. Captured user engagement was captured from Dec 2021 to Dec 2022 spanning the feasibility study period.

**Table 1.** Participant details from RPM dataset.

Patient ID	Site	Main illness	Gender	Age	Dates	Nurse Navigators
01	Site 2	HF	Female	79	12/2021-01/2022	NN1, NN2
02	Site 2	HF	Male	78	05 - 07/2022	NN1, NN2
03	Site 1	HF	Male	85	10 - 11/2022	NN3
04	Site 1	HF	Male	67	01 - 02/2022	NN3
05	Site 1	CRC	Male	71	04 - 05/2022	NN4, NN5
06	Site 1	CRC	Male	55	06 - 08/2022	NN4, NN6, PhD
07	Site 1	CRC	Female	66	11-12/2022	NN5, NN6, PhD

HF: heart failure; CRC colorectal cancer, NN: nurse navigator, PhD: nurse PhD candidate.

In the interview dataset, a total of eight nurses were interviewed; their mean age was 31 (25-39) and all but one were female. Included data was from three dyadic interviews and two individual interviews with nurses conducted after the feasibility study.

### Quantitative description nurse work from using the RPM intervention

Analysis of mixed data from the RPM dataset enabled a description of nurse work within the RPM intervention (Dignio Prevent). A total of 677 data strings were generated, each linked to notification or messages exchanged between patient-nurse dyads within the RPM application (mean data strings per patient: 96.7, SD: ±28.1, range: 54-133). Nurse response time to all notification types, was a mean of 26.8 hours (SD: ±47.4 hours, range: 1.2 minutes – 16.4 days). Data strings were mostly related to application notifications 72.6% (n=484), of which 46.1% (n=223) were labelled as safety notifications by Dignio Prevent. Data strings included text messages, 28.9% (n=146) between nurse and patient dyads, where patients (n=7) sent a total of 73 messages. The remaining data strings, 7% (n=47) had no RPM application notification flag created. These data strings contained information that ranged from system records of data editing, notes the nurses created (nurse documentation of phone calls to patients or details of patient reports), system documentation of video calls, and possible errors in the software (notification raised with no flag and not connected to data inputted).

### Qualitative description of nurse work convergence between data sets

Independent coding of the data sets converged and were integrated into three themes: (1) intervention technical and operational work, (2) digitally enabled care management and coordination, and (3) educational and relational work.

#### Theme 1: Intervention technical and operational work

**Figure 2.**
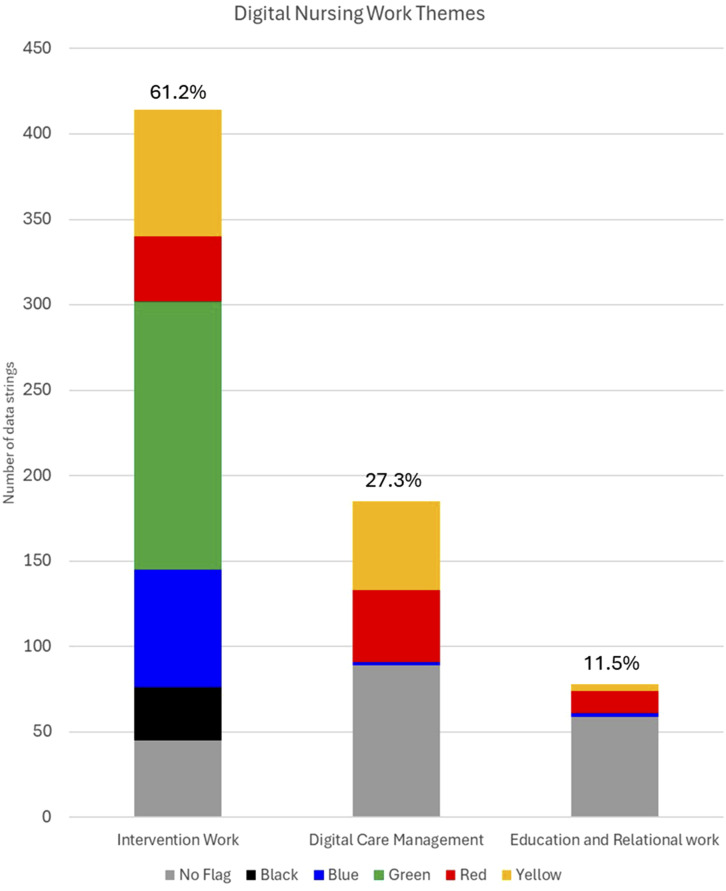
Digital nursing work themes: quantification of themes from the RPM dataset study. No flag: text message content, nursing follow-up or notes, Black: system notification, Blue: new patient message notification, Green: missing data notification, Red: severe safety notification, Yellow, moderate safety notification.

This theme identified in both datasets was that of work which related to technical and operational functionality of the interventional application. It was defined as any work required to ensure the digital platform was functioning and the patients were engaged with the intervention. Results from the RPM dataset demonstrated that the portion of work in this category was substantial 61.2% (n = 414 data strings) (Figure 2). Observed nurse actions included technical support, training participants in the use of the application, managing data errors, communications on technical issues, replacing failed devices/equipment, refining personalized safety parameters, and clearing of notifications. In the interview dataset nurses reported overall satisfaction with the intervention application they did raise concerns with some of the software functionality and their user interface.

“We had to click through an unnecessary number of steps. In addition, every feature on the platform was not available to us, so when I ‘played around’ in the platform, I have thought that some of the steps are unnecessary.” (Int 2: NN 3)

**Figure 3.**
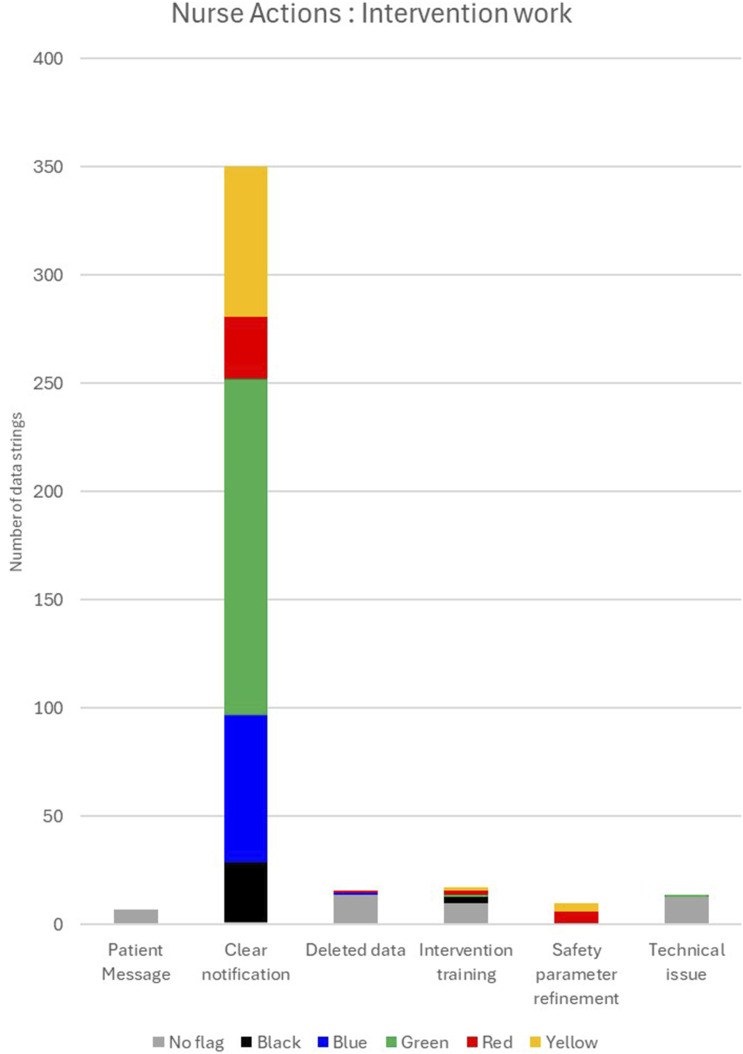
Nurse Actions by notification in theme1, identified in the RPM dataset. No flag: text message content, nursing follow-up or notes, Black: system notification, Blue: new patient message notification, Green: missing data notification, Red: severe safety notification, Yellow, moderate safety notification.

Results from the RPM dataset indicate that nursing actions in this work category regardless of flag colour were primarily (84%, n=350) related to clearing notifications (Figure 3). On examination in the more prevalent flags (Green, Yellow, and Red) there were observable times where this work was likely connected to possible refinements needed in application and safety parameter functionality. Frequently the safety flags had no documentation or were clinically evaluated as stable or expected changes (Table 2).

**Table 2.** Details on nurse actions related to most common system notifications in the theme, intervention technical and operational work.

​	Green: missing data	Red: safety	Yellow: safety
Application functionality	No documentation provided (n=89)	​	Low battery (n=32)
	Hospitalisation (n=30)		
	Data entered after deadline (n=24)		
	Technical support (n=11)		
	Patient messages (n=3)		
Safety parameter functionality	​	No documentation provided (n= 25)	No documentation provided (n= 35)
		Adjust safety parameters (n= 5)	Adjust safety parameters (n=4)
		Message sent to patient (n=3)	Message sent to patient (n=1)
		Intervention training (n=2)	Intervention training (n=1)
		Clinically stable (n=1)	Clinically stable (n= 1)
		Measurement errors (n=3)	​
Total Notifications	157	39	74

n represents number of data strings where this nurse action was observed.

These observations were triangulated with data from the interview dataset. Nurses reflected on how during the feasibility study they had to provide a considerable amount of technical and operational support outside of the traditional roles of a nurse (Additional Material 3).

“Sometimes when I called the support line, it took a while to find the right person, you had to talk to this person, and then you had to talk to that person because they supposedly knew better … they just kept referring me around.” (Int 5: NN 4)

Nurses described how this work was burdensome, and how the user interface made the work more difficult.

“Sometimes it was a bit cumbersome with those warnings, to remove them. You had to assess each one, even though there was nothing special to assess really. It was a bit knotty to go back and forth I think.” (Int 3: NN 5)

“I logged on in the morning. I had a quick check to see if there was anything I had to deal with immediately. If the tasks were small, I did them right away, but if it would take me a long time to complete, I had to do them when I had finished my other tasks [in the ward].” (Int 4: NN 7)

Nurses also described how the platform handled errors produced by the patient or the device in the measurement of clinical observations. Adding extra technical work for the nurses. Errors (false negatives) also occurred when patients missed the data submission deadline either because of a personal preference to submit in the evening or an inability to submit due to a hospitalization. While the amount of these occurrences appears low in the RPM dataset data (Table 2) it increased the nurse’s frustration as seen in interview dataset.

You had to check every single notification before you could remove them and there were lots of ‘nonsense’ notifications, for example reminders to do measurements, but I still had to check each notification before I could remove it. (Int 5: NN 4)

“I occasionally received measurement registrations twice, which generated twice the work. Not that it took me a long time, but I had to click on each notification, assess it, only to assess the exact same notification again.” (Int 3: NN 6)

#### Theme 2: Digitally enabled care management and coordination

The second theme identified across both datasets was digitally enabled care management and coordination. This theme was characterized by work related to assessing data input by patients, responding to that data, and engaging in interprofessional collaboration to ensure safety and provide clinically appropriate responses to changes in the participant’s condition. Analysis of the RPM dataset reported that this theme represented the second highest proportion (27% n=185) of nurse work (Figure 2). Observed nurse actions included clinical evaluation (stable, expected or unexpected change), clinical pathway impact (medication management, hospital admissions, referral to onward care teams), and interprofessional collaboration. Nurses, in the interview dataset, reported how the evaluation of the patient data input often was the tip of the iceberg for the work that needed to be confident in their clinical decision making.

“There were often alerts that the patients didn’t feel well, their blood pressure was low, their breathing was heavier. So, what could be the reason for that? Did they drink too much, eat too much? To make a good assessment, we had to ask them a lot of questions.” (Int 2: NN 8)

“If a patient sent me something I wasn’t sure of, I contacted the other nurse navigator. I also showed some of the pictures to the doctors, quite a few patients sent pictures of their wounds, whether they looked good or not.” (Int 5: NN 4)

**Figure 4.**
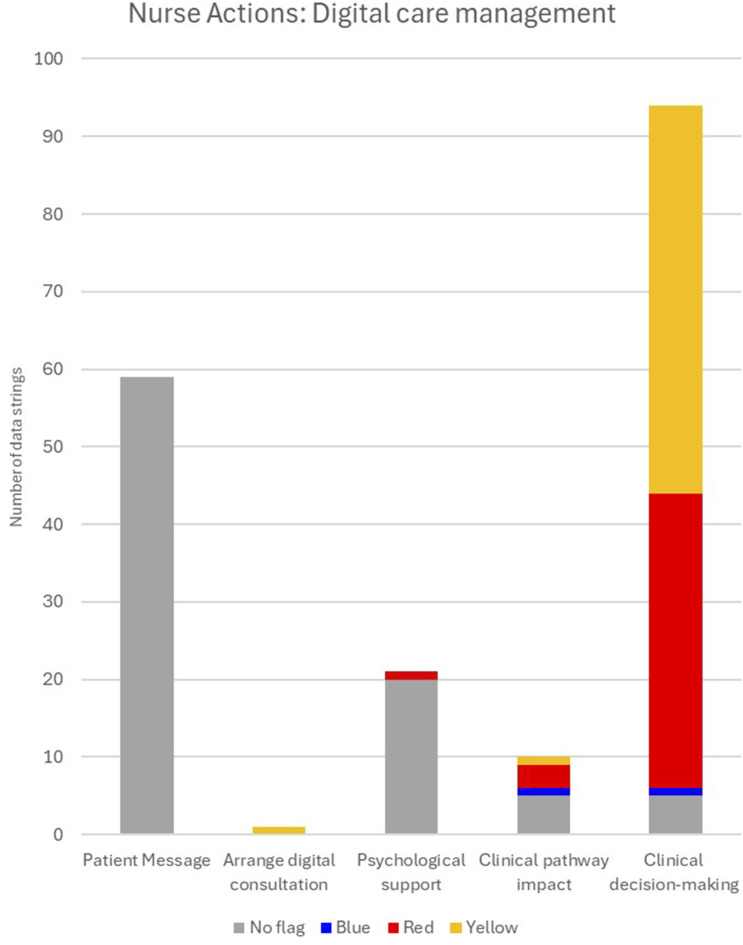
Nurse actions by notification in theme 2, identified in the RPM dataset. No flag: text message content, nursing follow-up or notes, Black: system notification, Blue: new patient message notification, Green: missing data notification, Red: severe safety notification, Yellow, moderate safety notification.

Results from the RPM dataset showed that nursing actions in this work category, regardless of flag colour, were mainly (51%, n=94) related to clinical decision-making (Figure 4). Interestingly, the data within the messages between nurses and patients (n=89) was similar to the portion of work identified around application safety flags (n=88). Text messages illustrated multiple occasions where nurses’ reassured patients on the nature of their symptoms and how their experiences were expected given their condition or symptoms.

“Hi! So good you found out. So nice that you follow along and let me know how you feel. We hope that you will be less bothered with palpitations, when you have been on increased dose of heart medication for a while.” (text message from NN1 to P01)

Frequently, the safety flags were assessed as stable or as an expected change (n=80), and only 16 times were safety flags documented as unexpected change.

“I had a patient [CRC] who lost about 2 kilos in 3 weeks. I wasn't worried, but he was very worried, and I understood that, so I called him up and made a plan with him. After that he was quite calm, so it turned out fine.” (Int 5: NN 4)

Similar to results in the RPM dataset, nurses mentioned that the clinical user interface would sometimes highlight data as a safety concern, however, after clinical evaluation, they determined that the only necessary action was to clear the notification, as the patient was fine.

“Some patients had really high scores, but when you contacted them, everything was fine. I called them up because I feared that they were sitting at home having an awful time, when they in fact were just fine.” (Int 3: NN 5)

The data generated by the nurse’s use of the application also identified when they had discussions with the patients or multi-disciplinary team (MDT) members (n=10) to confirm the clinical relevance of the data in the RPM. In MDT discussion there was documentation in the RPM platform on clinical consultations around a hospital admission, medication changes, or handover to the subsequent clinical team.

Results from the interview dataset supported these observations. Nurses reported how the platform provided them with data needed to evaluate a participant condition to then formulate a clinically appropriate response.

“We talked to each other about patients’ symptoms or their measurements, like, what could be the cause, what we could do, if we should do something, when to react, when we should talk to the patient, when we should talk to the doctor, we often discussed back and forth.” (Int 2: NN 3)

Nurses reported, since patient data could arrive at any time, this caused stress due to the limited time to manage RPM work, and it could be difficult to coordinate with medical doctors and other nurses.

What I found a bit challenging was, we paid extra attention to one of the patients’ weights. She often needed extra diuretics, which we had to discuss with the doctor. However, he didn’t always have the time. It was stressful because he didn’t have the time, and it was stressful for me to try and find the time too, because I had to finish my other assignments. (Int 4: NN 7)

The slow response rate by other clinicians also occurred with patients. Nurse management of poor responses, either from test messages sent or missing data, contributed to nurse stress and work.

“It could take a while to get answers to everything. If you ask them three questions, they only answer one of them. In order to get the full picture, you then have to ask again and again.”. (Int 2: NN 8)

#### Theme 3: Educational and relational work

The final theme identified in both datasets was educational and relational work. This theme was characterized in the RPM dataset across multiple nurse work categories including nursing documentation, education and engagement with patients, clinical evaluation, self-management guidance, communication between nurses and patients, and in MDT consultations to build a relationship that would facilitate the teaching and reinforcement self-management work. While this theme was only observed in 11% of the data strings (Figure 2), the nurses use of phone calls likely meant this work occurred outside of the RPM digital application.

“It was easier to call them and clarify the situation. Also, everyone has their phone with them wherever they go, but not everyone walks around with an iPad. It's actually faster too, I think, to clarify things on the phone. You get more detailed information than a message.” (Int 2: NN 8)

In the interview dataset, nurses recognized how the RPM platform (My Dignio and Dignio Prevent) facilitated their ability to build relationship with participants through digital consultations. In these relationships they were then able to use imputed patient data to guide and education patients on the self-management of their illness.

“I had one patient in particular who had a lot of questions. ‘'What can I do?', 'Can I go for a walk?' Questions about everyday activities.” (Int 4: NN 1)

“Some patients asked, when they noticed that their blood pressure was a little lower one day, whether or not that was normal. We usually advised them to wait it out, it wasn’t really that much lower, it just looks worse when it's below a 100 systolic than above 100. But really it isn’t that much of a difference.” (Int 4: NN 1)

Nurses also reflected on how the mutual engagement through the digital platform enabled them to provide additional psychological support to patients.

If they worried about symptoms, I explained it could be temporary and that it could be better the next day, or I simply just listened to what they had to say. (Int 2: NN 8)

**Figure 5.**
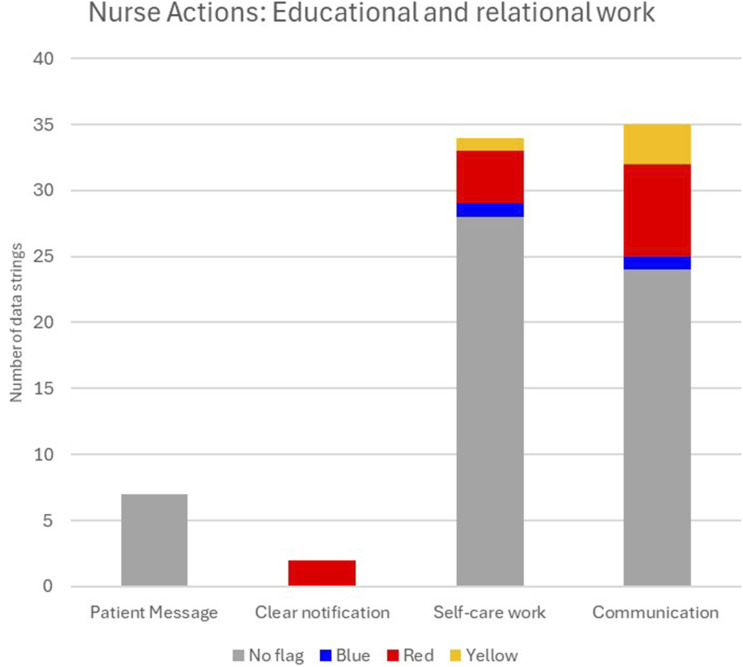
Nurse actions by notifications in theme 3, identified in the RPM dataset. No flag: text message content, nursing follow-up or notes, Black: system notification, Blue: new patient message notification, Green: missing data notification, Red: severe safety notification, Yellow, moderate safety notification.

Results from the RPM dataset highlighted how the nurse actions, in this theme, regardless of the flag colour, tended to be related to communication using text messaging, phone calls, and video calls (n=35) and self-management work (n=34) (Figure 5). Seventeen flags in this theme were labelled as safety (red or yellow), but nurses added documentation, with in the RPM application, demonstrating how these flags were a stable clinical measurement for the patients (n=6), or that the nurses had attempted conversations with the patient or MDT team members and were awaiting replies (n=11)

“Stable. Planning a conversation with a patient. No action”.

(Free text details from NN2 on Red flag for answer to daily symptom questions from P01).

“Have contacted a doctor regarding blood pressure, awaiting an answer.”

(Free text details from NN3 on red flag for Blood pressure, 93/56, from P03).

The nurse’s ability to follow along the patient experiences to facilitate similar evaluations and actions was reported in the interview dataset. In this study, the data labelled with a safety flag appeared to function more as a nudge to the nurses to evaluate the patient data and check what was really going on with the patient.

“If I saw that patients had lost a little weight, or if they had written on the questionnaire that they were restless or slept poorly, I often messaged them first and asked them about that problem. And quite often they didn’t need any help, only that someone acknowledged their problems.” (Int 5: NN 4)

“Made an appointment for a conversation with the patient, to hear a little more about how it's really going.” (Free text details from NN2 on yellow flag on weekly quality of life questions from P01).

## Discussion

The current study provides a novel method to evaluate the use of integrated data generated through nurse-patient dyad engagement with the RPM intervention. That data, alongside qualitative interviews provided an in-depth description of the digital nurse work. The proposed method along with the results and findings highlight how detailed evaluations can reveal clinical inefficiencies in RPM platforms. Modification based on such evaluations could improve the burden of this work and decrease barriers to implementation.^24^

The use of a convergent mixed methods design appears beneficial for comprehensively understanding the complex dynamics of nursing workload and decision-making in the context of an RPM intervention. The integration of system-generated mixed data and qualitative insights from interviews provided rich evidence that captured both objective workload metrics and subjective experiences of nurses engaged in RPM tasks. This blend of methodologies aligns with the principles outlined by Fetters, Curry,^33^ who emphasize the ability of mixed methods research to tackle intricate processes within healthcare settings.

The novel analysis method used here extends past usual measures, demonstrating areas where gaps in platform capabilities cause additional, sometimes burdensome, labour for nurses. It appears to identify areas for digital application improvement and emphasizes the significance of thorough clinical documentation. This method may be valuable given known challenges in transitioning digital applications from research to real-world settings, where technical issues and workflow misalignments often emerge.^2^ Digital health interventions, such as RPM are often evaluated with a narrow focus on patient outcomes and usability.^11^ This approach overlooks the clinical users, such as nurses, whose work is essential in succeeding with these interventions.^34^ While adherence, usability, and user interface are essential measures, they do not cover the entire breadth of work performed by clinicians required to sustain digital interventions.

The first theme identified in this study highlighted how RPM platforms may generate substantial invisible labour for nurses. The intervention aimed to boost patient self-management competence,^14,28^ it also necessitated significant technical and operational work from nurses; many of which fell outside traditional nursing roles. These included managing device failures, handling software issues, and clearing notifications, often with limited support or inefficient user interface. Our study reported high proportion of work related to notification management. The nurses’ reflections on task repetitiveness and lack of clinical relevance of many alerts, demonstrates how this method, applied in the current study, may inform platform refinements to improve usability for clinicians. The data revealed that many safety flags were clinically assessed as stable or expected changes, with only a small fraction requiring clinical action. While this suggests the platform was effective in prompting nurse review, it may also contribute to alert fatigue and unnecessary cognitive load.^35^ In America it has been recognized that the increasing use of digital tools to manage patients is contributing to physician burnout. The volume of data, system alerts and complex interfaces require substantial time and increase after work commitments.^36^ Nurses’ comments about needing to assess each notification, even when no action was required, reinforce the need for smarter alert systems that better differentiate between clinically significant and routine data, or even lend support to the body research supporting artificial intelligence as a mean to evaluate data input from patients.^37,38^

The second theme highlighted how digital nursing care may present significant challenges concerning clinical responsibility and decision-making. These problems were focused in key areas: (1) managing patient poor responses, and (2) integration of digital work into existing clinical pathways. Firstly, there will be a degree of patients who use RPM platforms whose response rate results in ethical quandaries relating to digital nurse work. For example, if patients do not respond to digital communications or failed to submit required data, as shown in the current study. Nurses become responsible for their action or inaction, which requires them to make decisions balancing patient autonomy with safety. According to the literature, nurses frequently face the obligation of following up on slow-responders, or non-responders and ensuring continuity of treatment, sometimes without defined protocols or adequate support.^19^ This can lead to ethical and professional quandaries, as digital communication is not always dependable or enough for therapeutic decision-making.

Secondly, the integration of digital tasks into existing clinical workflows can be challenging. Nurses described how the platform’s design sometimes hindered clinical efficiency. Without considering usability for clinical users, RPM applications may have design limitations which may increase the risk of having RPM becoming an additional burden.^2^ The unpredictability of patient data submissions also created stress for the nurses, particularly when coordination with other healthcare providers was required. While PRM platforms can provide nurses with the data needed to initiate these conversations, the lack of integration with broader clinical systems could sometimes make coordination difficult. This illustrates how RPM needs to be adequately supported by organizational structures to be successful.^24,35^

The observation of limited documentation in the RPM dataset around the final theme; educational and relational work was unexpected, but the theme was strongly observed in the interview dataset. It appears that nurse navigators used the platform, as a tool, to build relationships with patients. While considerable nurse work was performed their interactions often extended beyond the digital interface, with nurses preferring phone calls for more nuanced conversations. While RPM platforms can facilitate relational work, they must be designed to complement, not replace traditional modes of communications. Nurses in the current study described how they used patient-submitted data to guide conversations about symptoms, self-management, illness education, lifestyle choices, and psychological support. These interactions were both clinically relevant and emotionally supportive, helping patients feel heard and reassured. The platform’s ability to prompt these conversations, even when no clinical action was needed, highlights RPM’s potential as a tool for patient engagement and empowerment. Within the RPM dataset, the analysis method used demonstrated how most safety flags functioned more as prompts for relational engagements rather than indicators of clinical risk. The nurse navigators often used these flags as cues to check in with patients, clarify symptoms, and offer reassurance. That observation reinforces the concept that digital alerts should be designed not only for clinical triage but also to support the relationship between patients and nurse.^19^

Recognizing and resolving the invisible labour associated with RPM is critical for long-term adoption and scalability. Health systems should engage in training, technical assistance, and workflow redesign to ensure that digital duties are integrated in ways that benefit, not burden, nursing personnel.^39^ Finally, as this study highlights, RPM’s success is dependent on the recognition and support of nurses’ complicated, diverse labour in an ever-changing setting.

### Limitations related to methodological considerations

The aim of the paper was to further test an exploratory evaluation method developed on a single participant.^26^ As such, the data in this paper should be considered as illustrative as the data obtained from the feasibility study was not designed for this purpose. It is a possible example for how data generated from digital RPM’s usage could be used for refinement of these platforms to optimize their usability and integration into healthcare services. There may be gaps in the characterization of work nurses performed that were not captured in the RPM platform due to the retrospective nature of this work. The method used facilitates the possibility of detailed characterization of nurse work tied to application functionality. Prospective testing could determine the value of this technique within future research.

Secondary analysis of qualitative data can be valuable.^31^ However, this approach comes with several limitations. Firstly, the interviews were conducted for different research questions. Thus, the data collected may not fully align with this study’s aim. Secondly, the questions that were asked during the interviews were not specific to the current study aim, creating additional gaps in the current findings. Thirdly, the data may not have been structured or rich enough to support the coding framework chosen for the secondary data analysis. However, the independent alignment of themes by two authors (RCA and HBW), provides interesting insight into possible core components of the work of RPM digital nursing.

The integration of qualitative and quantitative data within a convergent mixed methods design can present significant methodologically complexities.^27^ The researcher’s independent analysis of different datasets before integration may heighten complexity. As evidenced in additional material 2 there was good alignment between researchers demonstrating convergence of the data which was then integrated. The commonalities observed in this exploratory work focused on testing an analysis method and could provide a foundation for future prospective research.^22^ By combining the datasets, nurses’ reflections shared in interview, added depth and greater understanding to the mixed method analysis of the RPM dataset. Through the integration of these different datasets and analysis methods, researchers can develop a clearer picture of the work associated with RPM tied to application functionality, ultimately leading to more robust technology that inform implementation strategies in healthcare systems.

Nurses were not instructed on their documentation within the intervention application. Their proactive nature meant they provided extra information in free text boxes in the RPM intervention, which was supplemental to their intervention training. Thus, the times where there was no documentation, does not reflect on nursing conduct, rather a limitation of this method and the retrospective nature of this research. By better understanding the competencies linked to digital nursing and the integration of RPM pathways into clinical workloads highlights the possibility of further refinements to make nursing documentation within such RPM application more efficient and streamlined. It underscores the need to develop digital nursing educational training programs^40^ and to develop robust digital nursing competencies.^41^

### Impact

Currently, there is a lack of detailed evaluation of clinical (nursing) work created by RPM interventions. This study aimed to test an innovative method that utilized data generated through patient and nurse dyad engagement with a digital RPM intervention. Using this method facilitated the detailed description of the proportion of work nurses performed tied to application functionality. The integration of different data sets facilitated detailed descriptions of three main themes of work performed by nursing: intervention technical and operational work, digitally enabled care management and coordination, and educational and relational work.

The presented evaluation method and analysis may have use as an informative foundation that can be integrated into future RPM intervention design. By enabling a prospective capturing of clinical work performed in RPM interventions data informed refinements to RPM platforms by software developers and researchers. It may provide the foundation for the development of digital nursing competencies and work, ensuring safe and efficient integration to healthcare systems.

## Conclusion

The novel evaluation method presented in the current study may provide an option which maximizes data generated in usage of an RPM platform to be integrated with interview data to characterise clinical workload impact. Using this method, digital nursing work using RPM platforms was identified and appears to aid in the care of people with chronic illnesses. There is likely room to improve the clinical interface of such platforms to reduce the burden of work on nurses. Future prospective research built around the present analysis method will be able to better define the type and proportions of clinical work in RPM platforms with subsequent implications on the adoption and implementation within healthcare systems.

## Supplemental material

Supplemental material - Describing digital nursing work in a remote patient monitoring application: Novel convergent mixed methods secondary analysis of feasibility trial data Supplemental material for Describing digital nursing work in a remote patient monitoring application: Novel convergent mixed methods secondary analysis of feasibility trial data by Rosalynn C. Austin, Bjørg Karlsen, Ingvild Morken, Sara Sudqvist, Aurora Selvik, Anne Marie Lunde Husebø, Hege B. Wathne in DIGITAL HEALTH

Supplemental material - Describing digital nursing work in a remote patient monitoring application: Novel convergent mixed methods secondary analysis of feasibility trial data Supplemental material for Describing digital nursing work in a remote patient monitoring application: Novel convergent mixed methods secondary analysis of feasibility trial data by Rosalynn C. Austin, Bjørg Karlsen, Ingvild Morken, Sara Sudqvist, Aurora Selvik, Anne Marie Lunde Husebø, Hege B. Wathne in DIGITAL HEALTH

Supplemental material - Describing digital nursing work in a remote patient monitoring application: Novel convergent mixed methods secondary analysis of feasibility trial data Supplemental material for Describing digital nursing work in a remote patient monitoring application: Novel convergent mixed methods secondary analysis of feasibility trial data by Rosalynn C. Austin, Bjørg Karlsen, Ingvild Morken, Sara Sudqvist, Aurora Selvik, Anne Marie Lunde Husebø, Hege B. Wathne in DIGITAL HEALTH

## Data Availability

Data will be made available on reasonable request.*

## Appendix

### Abbreviations

CRC: Colorectal cancer
HF: Heart failure
Int: Interview
MDT: Multi-disciplinary team
NN: Nurse navigator
QUAN: Quantitative
QUAL: Qualitative
RPM: Remote patient monitoring.
