# Supplementary material for: Describing digital nursing work in a remote patient monitoring application: Novel convergent mixed methods secondary analysis of feasibility trial data
Source: Digit Health. 2026 Jun 18;12:20552076261462734. doi: 10.1177/20552076261462734 (PMC13291451; doi:10.1177/20552076261462734)
Supplement: Supplemental material - Describing digital nursing work in a remote patient monitoring application: Novel convergent mixed methods secondary analysis of feasibility trial data [file sj-pdf-1-dhj-10.1177_20552076261462734.pdf]

RPM data set coding with exemplar quotes

Preliminary themes: **Nurse Work** with definitions	
	Exemplar Quote or Data string
**Assessment and Monitoring**: any nurse work related to the data participants entered in the RPM platform.	P01 input weight (86.8 kg) → Yellow flag → NN1 cleared notification commenting: “Expected weight loss”
	P02 input answer in daily HF symptom questions → Yellow flag → NN2 cleared notification commenting “stable situation, no action”
	P03 input blood pressure (93/56) → Red flag → NN3 cleared notification commenting: “Have contacted a doctor regarding blood pressure, awaiting answer”
	P04 input answer in weekly HF quality of life questions → Red flag → NN3 cleared notification commenting: “Notification values will be changed. Conferred with Dr regarding difficulties on setting good limit values on questionnaires”
	P05 input answer in daily CRC symptom questions → Yellow flag → NN4 cleared notification commenting: “some pain from the wound and some emptying problems, sent message for clarification”
	P06 input weight (87.9 kg) → Yellow flag → NN6 cleared notification and left no comment
	P07 input answer in weekly CRC symptom questions → Red flag → NN4 cleared notification commenting: “Score 1 of 5 points, leading to red alert, no alarming warning”
**Care Coordination**: any nurse work that led to the consultation of other clinical team members	P01 input answer in daily HF questions --? Red flag → NN2 cleared notification and commented “Phone call with patient, which indicates good “AT” and habitual symptom burden. Conferred with Dr, no further action required”
	P01 inputted blood pressure (146/100 mmHG) → Yellow flag → NN1 Cleared notification and commented “ Notified nurse at the heart failure outpatient clinic of persistent somewhat high blood pressure, as this is relevant in terms of increasing heart failure medication. Next check up at the heart failure clinic [date-within 7 days]”
	P07 message to nurse → Blue flag → NN4cleared the notification and commented “The patient is nearing the end of the follow-up [research study]. Given a number for the cancer phone until we have cancer days at [name of hospital ward], she is very happy”
**Digital consultation**: any form of digital (video or texts) between nurses and participants	“Hello, P01! Referring to our phone call today. I’ll call you tomorrow at about 15:30 if the scale still doesn’t work. Have a nice day, greetings NN”
	“Finished measuring today. Shape better than in a long time. Last night I slept all night and it’s actually the first time in a long time. It was a good feeling. Greetings P2”
	NN3 and P03 participated in a video call (46 seconds)
	Hi P03. How is your head today? I see that your blood pressure has been somewhat lower in the

	last two days than it has been before. Have you managed to drink enough during the day? Too low a fluid/water intake can contribute to both your headache and that your blood pressure is somewhat lower. Palpitations can be felt in several ways - including feeling your heart pounding hard in your chest, feeling like your heart skipping a beat or your heart getting an extra beat. Not everyone knows any of these things. I can call you today at 15.00, so we can have a chat. Is this true? Best regards, NN3”
	P04 inputted answer in daily HF questions → Red flag → NN03 commented “Talked to my passport. In a video call, this is his normal state as of now, he does not experience more wheezing/palpitations than usual. Patient takes their medications but experience reduced appetite as a result of the amount of tablets.”
	P06 text message “Hello struggling with fever and nausea today. Is this normal?” → PHD text response “Yes, it is normal, but if the fever rises, you should get in touch. Otherwise, try to drink well”
	“Hi P07, I've talked to Hege and you're up and running. Here you can ask questions or if you want to arrange a video call if you wish. Regards Follow-up NN5”
**Non-digital consultation**: phone calls or face to face conversations	Text message from P01 → Blue notification → NN2 cleared notification and commented “Phone call with guidance. Appointment for a new phone call tomorrow, if the scale does not work.”
	P01 blood pressure (144/100 mmHG) → Yellow Flag → NN2 cleared notification and commented “Phone call with passport. which indicates good AT and habitual sympathy burden. Conf. Dr. No further action is required.”
	New message from P03 → Blue notification → NN3 cleared notification and commented “Have talked to the patient, recommended to get enough fluids. Plan to confer with a doctor.”
	P04 pulse (46 BPM) → Yellow → NN3 cleared notification and commented “Carried out an already planned conversation with the patient. Pas. is asymptomatic with this pulse, he experiences it as normal.”
	NN4 Note → “Called P07 regarding change of settlement responsibility. P07 got info about stoma equipment when she had had a leak”
**Technical work**: non-nursing work related to the function of the intervention	System alert for P01 “Deleted measurement: Body weight 66 kg, date [date] time [time], equipment info missing. Deleted by NN1, date [date] time [time]. Reason: test.
	P02 → Green flag → NN1 cleared notification “missing weight” and commented “The patient has been admitted.”
	P03 → Green flag → Daily HF questions not answered. NN3 cleared notification cleared, no comment.
	P04 → Green flag → Low battery warning. NN3 cleared notification and commented “Sent message to patient. ‘Remind you of charging. I'll talk by phone tomorrow.’”

	P05 temperature (33C) → Red Flag → NN4 cleared notification and commented “Error measurement Testing”
	P06 new message → Blue flag → NN04 cleared notification, no comment provided
	PO7 new message → Blue flag → NN5 created “note” cleared notification and commented “NN4 IR has had a telephone conversation with the patient earlier today.”
Preliminary Themes: **Nursing Actions and Outcomes** with categories	
Code	Exemplar quotes and/or data strings
**Clinical evaluation**: actions related to nursing evaluation of the participant were further categorised with a nursing outcome: stable, expected change, unexpected change, arrange consultation, safety parameter refinement, psychological support, self-care monitoring, or self-care management.	*Stable:* PO1 answer in daily HF questions → Red flag → NN1 cleared notification and commented “Considers the answers to be okay despite the red notification”.
	Expected change: P04 answer in daily HF questions → Yellow flag → NN3 cleared the notification and commented “Improvement of symptoms”
	Unexpected change: P05 Answer in weekly CRC questions → Red flag → NN4 adds a note “Scores 4 on sleep, worry, fear” NN4 sends text message to P05 “Hi P05! I see you score something high on poor sleep and anxiety. How do you sleep? Is there anything I can contribute with in terms of fear and anxiety? Would you like a phone call? Regards, NN4”
	Arrange consultation: NN3 sent P03 a text message “Hi P03. I would like to have a video call with you to talk about your symptoms. Is this suitable for you at [time and date]]? I'll try to call you then. Can you possibly suggest another time if this should not be suitable? Best regards, NN3”
	Safety parameter refinement: P01 weight (83 kg) → Yellow flag → NN2 cleared notification and commented “Observed further. May consider adjusting the limit in the event of further weight loss”
	Psychological support: NN1 created a “follow-up note” for P02. “Called the patient as agreed today at 12.00. The patient indicates that he is in good shape and says that he thinks it is exciting and fun to take measurements and follow his form. Stated that he was somewhat anxious after going home, as he lay in the evenings and "felt a little too well" if, for example, he was short of breath. Reassured that this is normal and understandable after what he has experienced. Ask the patient to contact us either by message or phone if he has any questions for us.”
	Self-care-monitoring: NN4 text message to P06 “Hello! How are you doing? Is the swelling around the wound improving? Regards NN4”
	Self-care management: NN3 test message to P04 “Hi P04. I see that you have given a high score on swelling in the legs, dizziness and shortness of breath. How do you feel today? Have you talked to your GP about this? Best regards, NN3”
**Intervention administration:** actions related to the delivery of the intervention,	*Intervention training:* PO4 answer in daily HF question → Red Flag → NN3 cleared notification and commented “Exercise [test/training] together with the patient”

functionality of the platform, or data management. Nursing outcomes of these actions were further categorised as intervention training, technical issue, deleted data, or clear notification.	*Technical issue:* NN1 cleared notification → not tied to flag in the system, NN1 did not leave a comment
	Deleted data: System notification → P02"Deleted measurement: Blood pressure systolic 104 mmHg, diastolic 64 mmHg, [date, time], equipment info missing. Deleted by NN1, date: [date, time]. Reason: Test, training.
	Clear notification: P01 missing blood pressure measurement → Green flag → NN1 cleared notification and commented "Forgot to enter the end date, but that's okay now."
**MDT consultation:** actions related to the consultation of the wider clinical team for any reason. Nursing outcomes were further categorised as self-care monitoring, self-care management, safety parameter refinement, clinical handover, medication management, arrange consultation	*Self-care monitoring:* P01 answer in daily HF questions → Red flag → NN2 cleared notification and commented "Phone call with patient. which indicates good AT and habitual sympathy burden. Conferred with Dr [name] No further action is required.
	Self-care management: NN1 test message to P01 "Hi P01. So good to hear that it passed. It sounds right to do it that way. Have talked to a doctor here, and he says that if this happens again and it does not go away after you have taken Nitroglycerin (and it has worked a little), then you must contact the emergency room or 113. Keep up the good work you're doing! Happy New Year from NN2 and NN1."
	Safety parameter refinement: P3 answer in daily HF questions → Yellow flag → NN4 cleared notification and commented "Adjustment of limit values tomorrow after consultation with those responsible for the project and possibly a doctor. Pas. have generally the same symptoms daily. Swelling in the legs/around the waist will be followed up tomorrow due to stable weight today."
	Medication management: NN3 text message to P03 "Hi P03 I have talked to Dr. [name], she thinks the drug change you had made is perfectly fine. We will continue to try as you have done today. I'll call you again tomorrow to find out how it's gone. Regards NN3"
	Arrange consultation: P01 blood pressure (142/102 mmHG → Yelow flag → NN2 cleared notification and commented "Relatively stable; tendency to somewhat high BT. To be observed further. Scheduled conversation with patient and will confer with the responsible doctor in connection with this."
	Clinical Handover: NN4 text message to P07 "Hi P07! It is nearing the end of follow-up through this service [research study] unfortunately. Have you talked to my colleague PhD about picking up equipment? If not, I'll send her a message :-) I would also like to ask you if you feel the need to have any closing conversation with me on the phone tomorrow or Friday? Both me and nurse [name] have cancer days at [hospital ward] where we can support, inform and reassure those who need it. We have not received a business card yet but you can call on Wednesdays to no. [phone number] :-) Hope you have a nice December! Hear from you, warm regards NN4"
**Nursing documentation:** actions where the purpose was to record or document	*Digital consultation:* NN3 create a "note" for P03: "NN3, P03 participated in a video call. The call lasted 15 seconds."

nursing decisions. Nursing outcomes from these actions were further categorised as digital consultation, self-care monitoring, clear notification, stable, non-digital consultation, hospital admission, intervention training, deleted data, technical issue, clinical hand over	*Self-care monitoring:* P04 answer in daily HF question → Yellow flag → NN3 cleared notification and commented "Sent message to patient" NN3 text message to P04: Hi P04. I see you haven't answered the daily questionnaire since Friday. It would be nice if you want to answer that now. Are you okay? Do you still have swelling in your legs/around the waist? Best regards, NN3"
	Clear notification: P06 daily CRC questions not answered → Green flag → NN5 cleared notification and commented "everything in order"
	Stable: P01 Answer in g daily HF questions → Red flag → NN1 cleared notification and commented "no action"
	Non-digital consultation: P01 answer in weekly HF questions → Red flag → NN1 cleared notification and commented "Will call the patient during the first few days to discuss what the patient has reported."
	Hospital admission: P02 weight (75.1 kg) → Red flag → NN1 cleared notification and commented "Patient re-admitted"
	Intervention training: P04 blood pressure (105/58 mmHG) → Black flag → NN3 cleared notification and commented "Test/training"
	Deleted data: NN6 text message to patient "I also see there is a weight of 2077, I think this is also wrong."
	Technical Issue: P04 daily HF questions not answered → Green flag → NN3 cleared notification and commented "answered afterwards"
	Clinical handover: NN4 entered "note" for P07 "Patient has had stable temp and weight overtime, had 2kg weight loss but states good knowledge of measures to keep the weight. Pas has been offered a final conversation with me and given a number to the phone we use on Wednesdays for the cancer days that I and palliative nursing and cancer doctors are part of."
**Education and engagement:** Nursing action related to educating or encouraging engagement around their illness or the intervention. Outcomes of this action were intervention training, digital consultation, non-digital consultation, psychological support, self-care monitoring, and self-care management	*Intervention training*: P01 answer in daily HF question → Red Flag → NN1 cleared notification and commented "Test to educate the patient"
	Digital consultation: NN3 test message to P03 "Hi P03. Welcome home! I will call you tomorrow, [date], at [time] to arrange a video meeting. Remember to charge your tablet before then. Best regards, NN3."
	Non-digital consultation: P02 Answer in daily HF questions → Yellow flag → NN2 cleared notification and commented "Stable. Arranged conversation with the passport. [date]"
	Psychological support: NN4 text message to P05 "Hello! I'll call you after [date] to hear if it's going better with what you've ticked off today didn't go so well! Happy 17th of May P05, we'll talk soon :)"
	Self-care monitoring: NN3 text message to P04 "Hi P04. I see you haven't answered the daily questionnaire since Friday. Nice if you want to answer that now. Are you okay? Do you still have

	swelling in your legs/around the waist? Best regards, NN3"
	SC management: NN4 text message to P07 "That's very good to hear! Good thing they fixed delivery so quickly, they are very accommodating over at [medical supplies company]. Have a good weekend yes, regards NN4"
**Self-care education:** nursing action related to teaching participants self-care skills related to their personal experience of illness. Outcome of these actions included: digital consultation, self-care management, self-care monitoring, and medication management	*Digital consultation:* NN1 text message to P01 "Hi P01. We see that you lose weight nicely. That is expected and good. In the middle of next week, you are halfway through the project and we are wondering if you would like a video call with us, so that we can talk and answer any questions? Greetings NN1 and NN2."
	Self-care management: NN3 text message to P03 "Hi P03. I see that your blood pressure is still a little lower than it has been. Are you dizzy? Do you still have diarrhea? Have you managed to drink a little more water? Regards NN3"
	Self-care monitoring: NN2 text message to P02 "Hello, P02! So great that you have gotten started and got all your tasks done today. We recommend that you take your blood pressure, pulse and weight after you have been to the bathroom in the morning, but before you take medication and eat breakfast, so you have done it completely by the book today! The most important thing, however, is that you find a routine that works for you. Wish you a good weekend! Greetings NN2"
	Medication management: NN4 text message to P07 "Hello! It's good that it worked out! Drink plenty of water :-) Are you taking any movicol? If it is nice to soften now, you do not need to use it yet, but if there is less air and a harder consistency, movicol can be a good safety. Regards"
**Technical Work:** actions nurse took was related to the devices rather than clinical work. Outcomes of these actions included phone call or clearing notification.	*Phone call:* New message from P01 → Blue flag → NN2 cleared notification and commented "Phone call with guidance. Appointment for a new phone call tomorrow, if the scale does not work."
	Clear notification: P01 missing weight → Green flag → NN1 cleared notification and commented "The weight didn't work but is up and running again now."
