# Supplementary material for: Describing digital nursing work in a remote patient monitoring application: Novel convergent mixed methods secondary analysis of feasibility trial data
Source: Digit Health. 2026 Jun 18;12:20552076261462734. doi: 10.1177/20552076261462734 (PMC13291451; doi:10.1177/20552076261462734)
Supplement: Supplemental material - Describing digital nursing work in a remote patient monitoring application: Novel convergent mixed methods secondary analysis of feasibility trial data [file sj-pdf-2-dhj-10.1177_20552076261462734.pdf]

Table – short version

Research question	Preliminary themes	Categories	Themes	Alignment with Rosalynn
**To explore how the nurse navigators worked around the data in the digital platform** (or how do they interact with and utilize data in the platform)	**Assessments**<br>**+**<br>**Consultations (between NNs and doctors)**	**Interpreting and responding to data**<br>**+**<br>**Collaborating interprofessional**	**Digital-enabled care coordination**	**Care coordination**
**To explore what the nurse navigators did during patient consultations** (or what roles do they play)	**Information and education**<br>**+**<br>**Listening and reassuring**	**Guiding and educating patients**<br>**+**<br>**Providing emotional support**	**Relational and educational functions in RPM/digital consultations**	**Digital consultation**
**To explore what of the worked performed by the nurse navigators was not related to nursing** (or falls outside traditional nursing roles)	**Sorting out notifications**<br>**+**<br>**Replacing medical equipment**	**Managing system alerts and notifications**<br>**+**<br>**Handling equipment logistics**	**Technical and operational support**	**Technical work**

Table 1: Theme 1

Meaning units	Preliminary themes	Categories	Themes
Sometimes we were like; Oh, there are red flags. We have to check what is going on here ... but then it turned out to be nothing. However, there could be a need to clarify what the patient really meant with his answers ... (Int 1: NN 1)<br><br>I checked each morning to get a quick overview of how things were. I didn't necessarily deal with alerts and things like that, but I could see if there was anything alarming. (Int 1: NN 1)<br><br>There were often alerts that the patients didn't feel well … their blood pressure was low … their breathing was heavier. So, what could be the reason for that? … did they drink too much … eat too much? To make a good assessment, we had to ask them a lot of questions. (Int 2: NN 2)<br><br>We were typically looking for the combination of heavier breathing, weight gain and increase in blood pressure (Int 2: NN 2)<br><br>I logged on in the morning. Then I had a quick check to see I there was anything I had to deal with immediately. If the tasks were small, I did them right away, but if it would take me a long time to complete, I had to do them when I had finished my other tasks.(Int 4: NN 7)<br><br>I would have liked to have blood pressure, weight and all of that on the same page ... that I didn't have to click on different features every time. I want to see all the different at the same time ... maybe in a small table, because I consider the different parameters together. (Int 2: NN 2)<br><br>We reached out to them when we noticed that their score was high and they had ticked off that they were in worse shape. We often sent them a message first, but if we didn't get an answer, or the answer was too short and we felt we needed more, we called them. (Int 2: NN 2)<br><br>Some patients had really high scores, but when you contacted them, everything was fine. I called them up because I feared that they were sitting at home having an awful time, when they in fact were just fine. (Int 3: NN 4)<br><br>If we send a message to one of the patients, we have no way of knowing if they see it the same day or the next day. To see if they had answered, we had to go in and check ... I often checked every fifteen minutes, but it could be hours before we received any answers. (Int 2: NN 2)<br><br>Some of the older patients didn`t write particularly long messages. So, it could take a while to get answers to everything. If you ask them three questions, they only answer one of them. In order to get the full picture, you then have to ask again and again. (Int 2: NN 2)<br><br>We wrote notes ... for example, when I talked to the patient about increasing their diuretics, I made a note in the Dignio platform. (Int 4: NN 6)<br><br>I wrote down notes every time. To each notification, I wrote a small note about what we agreed on. (Int 2: NN 2)<br>I could tell the doctor; 'The patients' blood pressure is decreasing; can we have a chat about that at the end of the day?' Then we reflected whether we should change the limit values, or we looked at the symptom reporting together. (Int 1: NN	**Assessments**	**Interpreting and responding to data**	**Digital-enabled care coordination**

1)<br><br>It was easy to get an overview picture I could show to the doctor. Even though he [the doctor] wasn't as familiar with the patient as I was, it seemed like it was easy for him to get a quick overview, and enough to be able to make decisions. (Int 1: NN 1)			
We talked to each other about patients' symptoms or their measurements … like, what could be the cause, what we could do, if we should do something, when to react, when we should talk to the patient, when we should talk to the doctor … we often discussed back and forth. (Int 2: NN 3)<br><br>What I found a bit challenging was … we paid extra attention to one of the patients' weight. She often needed extra diuretics, which we had to discuss with the doctor. However, he didn't always have the time. It was stressful because he didn't have the time, and it was stressful for me to try and find the time too because I had to finish my other assignments. (Int 4: NN 7)<br><br>I would check during my day shift to see if anything was alarming, but sometimes I didn't have time to respond to the alerts. The I would ask my fellow nurse navigator, when she started her evening shift, if she could respond to the alerts. (Int 4: NN 5)<br><br>If a patient sent me something I wasn't sure of, I contacted the other nurse navigator. I also showed some of the pictures to the doctors ... quite a few patients sent pictures of their wounds, whether they looked good or not. (Int 5: NN 8)	**Consultations (between NNs and doctors)**	**Collaborating interprofessional**	**Digital-enabled care coordination**

Table 2: Theme 2

Meaning units	Preliminary themes	Categories	Themes
If they worried about symptoms, I explained it could be temporary and that it could be better the next day … or I simply just listened to what they had to say. (Int 2: NN 2)<br><br>I don't think we communicated that much about health issues … it was more about practical things in the chat. If I had questions and wanted to know how the patient really was doing, I used the phone. (Int 3: NN 5)<br><br>Video was cumbersome to use, because you couldn't just give them a call … it had to be scheduled. So, we talked on the phone instead. (Int 2: NN 2)<br><br>It has been easier to call them and clarify the situation … and also, everyone has their phone with them wherever they go, but not everyone walks around with an iPad. It's actually faster too, I think, to clarify things on the phone. You get more detailed information than a message. (Int 2: NN 2)<br><br>We had to provide one of the patients with some extra follow-up because of her medication. She received care at home, so we called both her and the home care service to provide them with information about taking extra diuretic at home. (Int 4: NN 6)<br><br>Some patients had specific questions, like 'Can you find out when my next appointment is', or they needed answers about wound tests. But we also provided them with more general feedback, like 'good job', or 'keep up the good work'. (Int 4: NN 7)<br><br>I had one patient in particular who had a lot of questions. "What can I do?', 'Can I go for a walk?' Questions about everyday activities. (Int 4: NN 6)<br><br>It was often the one in poorest condition who had the most questions ... and often the conversations took place over the phone and not by message. (Int 4: NN 6)<br><br>Some patients asked, when they noticed that their blood pressure was a little lower one day, whether or not that was normal. We usually advised them to wait it out .. it wasn't really that much lower … it just looks worse when it's below a 100 systolic than above 100. But really it isn't that much of a difference. (Int 4: NN 6)	**Information and education**	**Guiding and educating patients**	**Relational and educational functions in RPM/digital consultations**
I could see that the patients scores were a bit up and down. However, they never scored particularly high on sadness and depression. But I nevertheless talked to them and asked them how they were feeling. (Int 3: NN 4)<br><br>I think they [the patients] appreciated getting a call … it was nice for them to hear a voice and talk a little. Also, they were able to learn more about their body, such as their blood pressure. They really gained more knowledge. (Int 4: NN 7)<br><br>I had a patient who lost about 2 kilos in 3 weeks. I wasn't worried, but he was very worried ... and I understand that, so I called him up and made a plan with him. After that he was quite calm, so it turned out fine. (Int 5: NN 8)<br><br>If I saw that patients had lost a little weight, or if they had written on the questionnaire that they were restless or slept poorly, I often messaged them first and asked them about that problem. And quite often they didn't need any help … only that someone acknowledged their problems. (Int 5: NN 8)	**Listening and reassuring**	**Providing emotional support**	**Relational and educational functions in RPM/digital consultations**

Table 3: Theme 3

Meaning units	Preliminary themes	Categories	Themes
It was easy to use I think … but we had to press a lot back and forth to enter the different features on the menu, but once you learned where everything was, it was easy. (Int 2: NN 2)<br><br>It [the platform] is slightly worse on the mobile version. On my phone, I have to scroll a lot to get a clear view, and that's a bit cumbersome if you just want to check something quickly. I prefer to do it on my computer, because the layout on the phone is too poor. (Int 2: NN 2)<br><br>Sometimes I logged on to the platform on my phone, only to see that there was nothing special. But you are not able to click out notifications on the phone ... you can't remove them. You can send messages and stuff, but you can't remove the notifications. So, then I had to log in to the computer … (Int 5: NN 8)<br><br>We have to click through an unnecessary number of steps. In addition, every feature on the platform were not available to us, so when I have been 'playing around' in there, I have thought that some of the steps are unnecessary. (Int 2: NN 3)<br><br>Sometimes it was a bit cumbersome with those warnings ... to remove them. You had to assess each one, even though there was nothing special to assess really. It was a bit knotty to go back forth I think. (Int 3: NN 4)<br><br>Particularly two issues that stood out: One was that the measurements were not transferred automatically to the tablet … even though everything looked okay and we double-checked. In these cases, everything had to be entered manually. There was also some discrepancies in patients' weight, in which case we had to replace the scale. (Int 2: NN 2)	**Sorting out notifications**	**Managing system alerts and notifications**	**Technical and operational support**
I was not able to have video calls with one of my patients … it was impossible. First I tried with one tablet … I added it, but it didn't work. Everything seemed perfectly fine on the tablet itself, but there was no sound. Then I tried to connect a new one, and it was exactly the same with that. (Int 2: NN 3)<br><br>Sometimes when you wanted to clear out a notification, you had to click several times to make it go away. There could be a message from the patient, but somehow you couldn't click on it ... you had to do it a few times before it went away.(Int 3: NN 5)<br><br>You had to check every single notification before you could remove them … and there were lots of 'nonsense' notifications', for example reminders to do measurements, but I still had to check each notification before I could remove it. (Int 5: NN 8)<br><br>I occasionally received measurements twice, which generated twice the work. Not that it took me a long time, but I had to click on each notification, assess it, only to assess the exact same notification again. (Int 3: NN 5)<br><br>The only thing I thought was a bit annoying was that you couldn't clear out and respond to several notifications at the same time. You had to click on each individual notification. That was a bit cumbersome. (Int 3: NN 4)<br><br>We had a patient who struggled a bit with usernames and passwords. But we were able to resolve everything over the phone … created a new password. We also had an iPad we were not able to unlock. It was stuck in a former patient's platform.<br><br>There could be seven notifications when I logged in, but I only had to care about two of them. (Int 5: NN 8)	**Replacing medical equipment**	**Handling equipment logistics**	**Technical and operational support**

One patient was unable to write messages. I called the support line two or three times, and we tried to update the tablet through their system, but she was still unable to write. (Int 5: NN 8)<br><br>Sometimes when I called the support line, it took a while to find the right person … you had to talk to this person, and then you had to talk to that person because they supposedly knew better ... they just kept referring me around. (Int 5: NN 8)			
