# Supplementary material for: Describing digital nursing work in a remote patient monitoring application: Novel convergent mixed methods secondary analysis of feasibility trial data
Source: Digit Health. 2026 Jun 18;12:20552076261462734. doi: 10.1177/20552076261462734 (PMC13291451; doi:10.1177/20552076261462734)
Supplement: Supplemental material - Describing digital nursing work in a remote patient monitoring application: Novel convergent mixed methods secondary analysis of feasibility trial data [file sj-pdf-3-dhj-10.1177_20552076261462734.pdf]

Final map of themes, categories, and meaning units

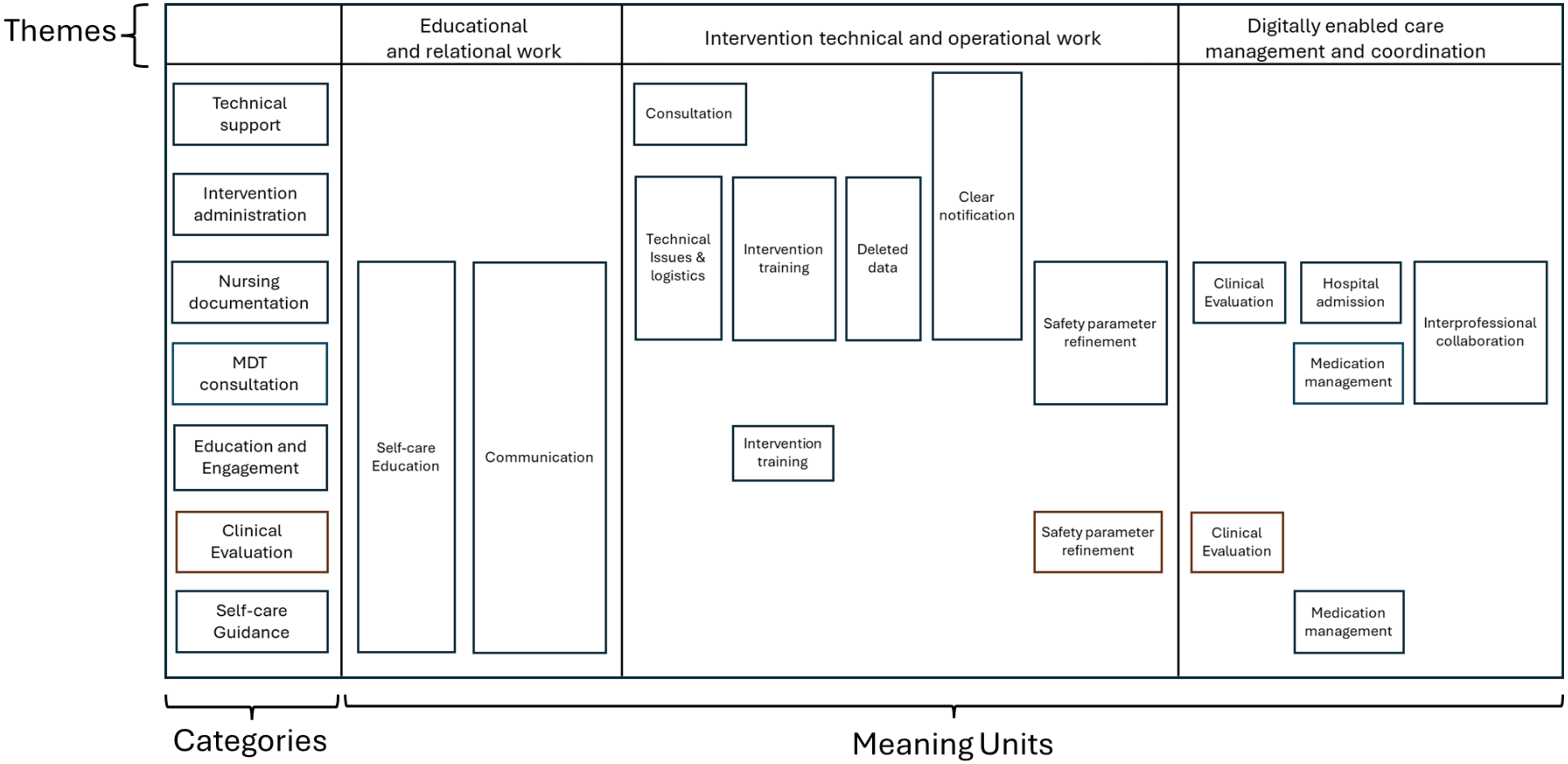

Integration of Phase 1 & 2 coding/themes

Themes

Final Theme	RPM dataset	Interview dataset
Digitally enabled care management and coordination	Monitoring management	Digital-enabled care coordination
Educational and relational work	Self-care education and reinforcement	Relational and educational functions in RPM/digital consultations
Intervention technical and operational work	Intervention work	Technical and operational support

Categories

Final categories	RPM dataset	Interview dataset
Assessment, interpretation, and response to RPM data	Nursing documentation, clinical evaluation, self-care guidance, patient messages	Interpreting and responding to data
Interprofessional care coordination	MDT consultation, clinical evaluation and outcomes, self-care guidance	Collaborating interprofessional
Communication, guidance, and education of patients	Consultations, nursing documentation, MDT consultation, education and engagement, clinical evaluation, self-care guidance, patient messages.	Guiding and educating patients
Emotional support	Consultations, patient messages, psychological support	Providing emotional support
Management of application notifications and parameters	Consultations, intervention administration, data management (delete data, error reporting), clear notifications	Managing system alerts and notifications
Intervention logistics	Technical issues and logistics, intervention training	Handling equipment logistics

Meaning Units

See supplemental material 1 and 2 for exemplar quotes
